# Treating prolonged grief disorder with prolonged grief-specific cognitive behavioral therapy: study protocol for a randomized controlled trial

**DOI:** 10.1186/s13063-018-2618-3

**Published:** 2018-04-20

**Authors:** Rita Rosner, Eline Rimane, Anna Vogel, Jörn Rau, Maria Hagl

**Affiliations:** 1 Department of Psychology, Catholic University Eichstätt-Ingolstadt, Ostenstr. 26, 85071 Eichstätt, Germany; 2 Coordinating Center for Clinical Trials of the Philipps University of Marburg, Karl-von-Frisch-Straße 4, D-35043 Marburg, Germany

**Keywords:** Prolonged grief disorder, Complicated grief, Integrative cognitive behavioral therapy, Present-centered therapy, Adults, Randomized controlled trial

## Abstract

**Background:**

Prolonged grief disorder (PGD) has emerged as a well-defined and relatively common mental disorder that will be included in the upcoming revision of the *International Classification of Diseases*. Recent trials with grief-specific, mostly cognitive behavioral interventions for patients with a clinically relevant diagnosis of PGD showed large effect sizes. However, a small trial suggested that non-specific behavioral activation might suffice to improve PGD. So, more evidence for the relative efficacy of grief-specific treatments is needed, as is more research on the predictors of treatment success. The purpose of the proposed trial is to evaluate a newly developed and successfully pilot-tested, prolonged grief-specific, integrative cognitive behavioral therapy (PG-CBT) compared to an active yet unspecific treatment, present-centered therapy (PCT).

**Methods:**

In a multicenter, randomized controlled trial with 204 adults with a primary diagnosis of PGD, PG-CBT is compared to PCT, assuming the superiority of PG-CBT. Both treatments consist of 20 to 24 individual sessions, with an overall treatment length of about 6 months. The primary outcome, grief symptom severity, is assessed by blinded interviewers 12 months after randomization. Secondary outcomes are grief symptom severity at post treatment, in addition to self-reported overall mental health symptoms, depressive and somatoform symptoms at post treatment and 12 months post randomization. Possible moderators and mediators of treatment success are also explored.

**Discussion:**

The trial is designed to avoid bias as much as possible (stratified randomization performed independently, blinded outcome assessment, intention-to-treat-analysis, balanced treatment dose, continuous supervision, control for allegiance effects) thereby enhancing internal validity. At the same time, some aspects of the trial will ensure clinical relevance (recruiting at outpatient clinics that are part of routine health care and keeping exclusion criteria to a minimum). Since the trial is powered adequately for the primary outcome, all secondary analyses including moderator analyses are exploratory by nature. The results will extend the knowledge on efficacious treatment of PGD and its predictors.

**Trial registration:**

German Clinical Trials Register, ID: DRKS00012317. Registered on 6 September 2017.

**Electronic supplementary material:**

The online version of this article (10.1186/s13063-018-2618-3) contains supplementary material, which is available to authorized users.

## Background

Prolonged grief disorder (PGD) has emerged as a well-defined mental disorder, distinguishable from major depression and posttraumatic stress disorder (PTSD) or other stress-related disorders [1]. Core symptoms are intense yearning and preoccupation with the deceased; reactive distress symptoms, such as feeling stunned or shocked by the loss; avoidance of reminders of the reality of the loss and emotional numbing, and social/identity disruption, for instance feeling detached or finding it difficult to trust other people [2, 3]. PGD will be included as a diagnosis in the 11th revision of the *International Classification of Diseases* (ICD-11), with slightly different criteria to those of its counterpart in the *Diagnostic and Statistical Manual of Mental Disorders, Fifth Edition* (DSM-5; [4]), the “persistent complex bereavement disorder.” Concerning prevalence, a recent meta-analysis that included 14 studies reported a pooled prevalence of 9.8%, i.e., one out of 10 non-violently bereaved adults might develop PGD [5].

PGD has been found to be associated with deteriorated health [6] and increased suicidality [7]. Comorbidity was high in treatment-seeking samples [8]. Most randomized clinical trials (RCTs) reported depression and PTSD as comorbid disorders and/or depressive, anxiety and posttraumatic stress symptoms as secondary outcomes (e.g., [9–11]). In our pilot trial, 54% of PGD patients were diagnosed with some kind of somatoform disorder [12]. At the present time, we are not aware of any other study that reports on somatoform comorbidity in PGD outpatients. And while there is growing evidence that cognitive behavioral interventions are highly effective for PGD, effect sizes were somewhat lower for comorbid conditions (e.g., [12, 13]). Thus, existing grief treatments seem to be specific.

Meta-analyses on the efficacy of grief treatment indicate that a formal diagnosis of clinically impairing prolonged grief is highly relevant. Whereas non-selective interventions for the bereaved were – in sum – ineffective, psychotherapy with individuals suffering from clinically relevant prolonged grief symptoms showed at least moderate effect sizes (e.g., [14, 15]). The latest meta-analysis for controlled trials found a significant but heterogeneous mean effect size of 0.53 for the treatment of PGD, based on five studies [15]. Since then, newer trials with grief-specific cognitive behavioral interventions in PGD showed better effects in diverse settings and populations [9, 12, 16, 17]. In a pilot trial, we evaluated a newly developed integrative cognitive behavioral therapy (PG-CBT) with 51 bereaved adults with PGD. We found a large effect of *d* = 1.32 compared to the waiting-list control group [12].

Nonetheless, more evidence is needed. While at least 25 RCTs with bereaved adults have been published since 2008 (the above-cited meta-analyses searched up to the year 2007), no more than 10 studies screened for either PGD as a diagnosis or for clinically relevant prolonged grief symptoms. In addition, some of these studies suffered from methodological problems (e.g., small sample sizes, non-blind evaluation of the primary outcome, or no stratification for crucial variables). For example, when it comes to sample size, most studies should be considered as mere pilot trials, with 25 or fewer participants randomized per group [11, 12, 18–20]. Only three recent trials evaluated large enough samples [9, 16, 17]. These three trials were the only ones with blind outcome assessment, too. Altogether, about half of the trials since 2008 tested against waiting list, which is also true for ongoing trials registered on the International Clinical Trials Registry platform.

Apart from insufficient evidence for the relative efficacy of PGD treatments, knowledge of predictors of treatment success is also incomplete. In epidemiological research, there is conflicting evidence for female gender and older age as predictors of prolonged grief, but robust evidence for losing a child or one’s spouse [21–23]. When it comes to treatment outcomes, the findings are even less clear. One study could not confirm any moderator [16], while another [24] found that a lower educational level and losing a child or partner predicted worse outcomes whereas initial levels of comorbidity did not. In our pilot study, patients seeking additional treatment after post assessment had a higher probability of having been diagnosed with a somatoform disorder at baseline [25]. Although somatization improved significantly from pre to post, there was no significant difference between the treatment group and the waiting-list group (in contrast to depressive symptoms; [12]). Apart from somatoform symptoms and other baseline comorbidity that should be addressed in therapy, several other possible predictors of treatment outcome warrant more research. For example, dysfunctional cognitions, grief-related avoidance, rumination and worry have been shown to predict poorer adjustment to loss in longitudinal studies (e.g., [26]) and might mediate the therapy outcome (see also [24]).

### Objectives of the current trial

The major goal of this trial on PROlonged GRIef Disorder – PROGRID – is to substantiate the positive results of our pilot-tested intervention [12] while remedying the methodological shortcomings of the pilot trial: (1) PGD symptoms are assessed in a structured interview by independent blind raters, (2) additional attention is paid to comorbidity and somatic symptoms in particular, (3) further predictors of treatment outcome are addressed as well as therapeutic process and change trajectories. Finally (4), we compare our intervention with an active, yet unspecific, treatment in order to evaluate the relative efficacy of grief-specific cognitive behavioral therapy (CBT). While one trial suggested that non-specific behavioral activation might suffice to improve PGD [11], Bryant and colleagues [9] showed that individual exposure sessions enhanced effectiveness, pointing to the relevance of some kind of emotional processing.

The experimental treatment, PG-CBT, is an integrative cognitive behavioral approach that includes structured exposure and cognitive restructuring. Altogether, PG-CBT consists of 20 individual sessions (plus four optional sessions) within 6 months of manualized treatment. We chose present-centered therapy as the comparator (PCT; [27, 28]). PCT was developed as a control condition for non-specific treatment factors such as empathic listening and therapeutic support in treatment trials evaluating CBT in PTSD. It achieved moderate to high pre- to post-treatment effect sizes for primary outcomes and was well tolerated (e.g., [29–31]). A recent meta-analysis, including six trials, found PCT to be slightly inferior to other therapies regarding PTSD symptom severity, but equally effective concerning secondary outcomes [32]. PCT does not include any trauma-focused components (i.e., exposure or cognitive restructuring of dysfunctional beliefs) but some basic components of behavioral therapy, i.e., education about the connection between posttraumatic symptoms and daily problems, and fostering problem-solving skills, including a daily diary. Thus, PCT is the ideal control condition for attention, support and problem solving, and suitable for PGD. In addition, it resembles the mostly supportive approach taken with grieving patients in usual care or in self-help groups.

Because of its integrative and comprehensive approach and based on the results from the pilot trial, we expect PG-CBT to be found to be not only superior to PCT regarding prolonged grief symptoms, but also regarding all secondary outcomes. Furthermore, the trial adds to existing knowledge by exploring possible moderators (e.g., age and somatoform comorbidity at baseline) and mediators of treatment outcome, in particular the role of grief-related avoidance, rumination and dysfunctional cognitions concerning the loss.

## Methods

### Trial design

**Fig. 1:**
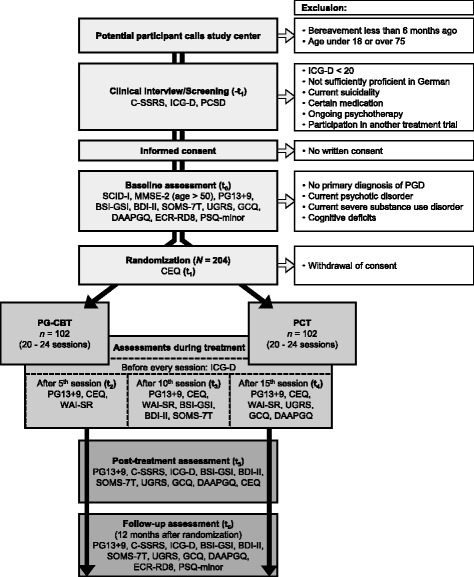
Participant flow. *CEQ* = Credibility/Expectancy Questionnaire; *C-SSRS* = Columbia-Suicide Severity Rating Scale; *BDI-II* = Beck Depression Inventory; *BSI-GSI* = Global Severity Index (Brief Symptom Inventory); *DAAPGQ* = Depressive and Anxious Avoidance in Prolonged Grief Questionnaire; *ECR-RD8* = Experiences in Close Relationships – Revised (short version); *GCQ* = Grief Cognitions Questionnaire; *ICG-D* = Inventory of Complicated Grief; *MMSE-2* = Mini-Mental-State-Examination; *PCSD* = Perception of Circumstances Surrounding the Death Scale; *PCT* = Present-centered therapy; *PG13+9* = Interview for Prolonged Grief-13, extended version; *PG-CBT* = integrative cognitive behavioral therapy for prolonged grief; *PSQ* = Pain Sensitivity Questionnaire; *SCID-I* = Structured Clinical Interview for DSM-IV Axis-I Disorders; *SOMS-7T* = Screening für Somatoforme Störungen (Screening for Somatoform Disorders); *UGRS* = Utrecht Grief Rumination Scale; *WAI-SR* = Working Alliance Inventory – self-report

In this multicenter, rater-blinded RCT with two parallel groups, the experimental treatment condition, PG-CBT, is compared to an active control group, PCT, assuming the superiority of PG-CBT after approximately 6 months of treatment (20 to 24 individual sessions), measured 12 months after randomization. Randomization is performed independently by a Clinical Trials Coordination Center (CTCC), employing block randomization with randomly varying block sizes stratified by study center and type of kinship (child vs. other). See Fig. 1 for participant flow through the study. The study protocol was written in accordance with the SPIRIT 2013 Statement (Standard Protocol Items: Recommendations for Interventional Trials; see [33]; for the SPIRIT Checklist see Additional file 1).

### Study setting and recruitment

Treatment is offered at four university outpatient mental health clinics in four German cities, Frankfurt, Ingolstadt, Marburg and Leipzig. Treatment-seeking individuals are routinely screened for having experienced the loss of a significant other. Additional recruitment efforts will include a study website, advertisements in public and social media (e.g., websites for bereaved persons, self-help forums), radio interviews, flyers in family practices, health and community centers, or churches, and informing general and mental health practitioners via mailings, as well as via talks and publications in the specialized press.

### Participants and eligibility criteria

Treatment-seeking adults, aged 18 to 75 years, with sufficient cognitive and German language skills, who provide written informed consent, will be included. Eligible participants must meet the criteria of a primary diagnosis of PGD as assessed in the PG-13 interview (Interview for Prolonged Grief-13, see below). If patients are taking antidepressant medication, the treatment regime needs to be stable for at least 4 weeks before joining the trial. Exclusion criteria are: (1) current psychotic or severe substance use disorder, or acute suicidality; (2) ongoing psychotherapy; (3) participation in another treatment trial; and (4) continuous treatment with benzodiazepines, antipsychotics, or opioids.

### Sample size

Given the large effect size found in our pilot study (Cohen’s *d* = 1.32) for comparing PG-CBT to a waiting-list condition [12], and the smaller yet substantial effect sizes found in studies comparing a grief-specific treatment with less specific (0.37; [34]) or supportive control groups (0.40; [13]), the assumption of an effect size of at least *d* = 0.42 seems reasonable when comparing the integrative, grief-specific PG-CBT to the unspecific PCT control condition. Therefore, power calculation for the primary endpoint of this trial is based on the assumption that PG-CBT is more effective, if a minimal clinical relevant difference of 5.5 points (PG-13 symptom severity) in mean change between baseline and 12-month follow-up could be shown between the groups. We expect the variance of the PG-CBT group to exceed that of the pilot study. The assumed *SD* = 13 is reduced by the factor [(1 – *r*^2^)]^1/2^ to a baseline-covariate adjusted *SD* = 12.4 with correlation of *r* = 0.3 in this multicenter study with a 12-month follow-up (pilot trial after approximately 6-month follow-up: *r* = 0.4). A total sample size of *N* = 162 is needed to detect the minimal clinical relevant difference between the two groups with a power of 0.80 and an alpha of 0.05. We aim to recruit 204 patients to allow for an estimated dropout rate of 20%.

### Procedure

**Fig. 2:**
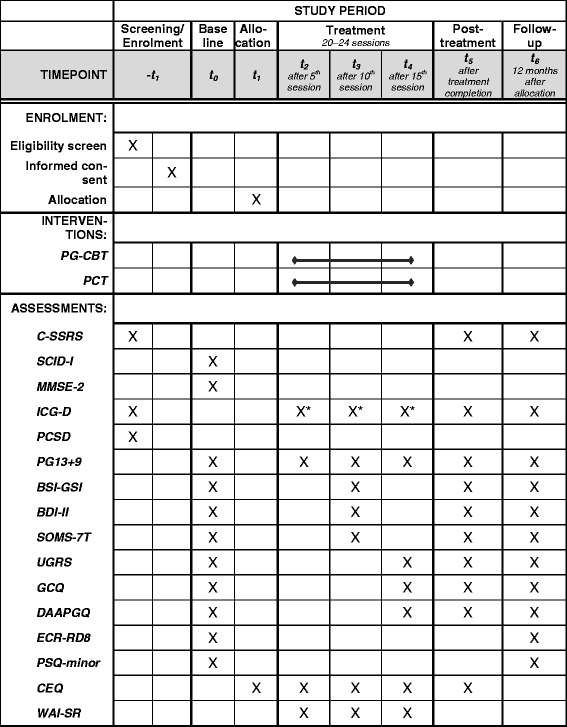
Schedule of enrollment, interventions, and assessments (SPIRIT Figure)*. CEQ* = Credibility/Expectancy Questionnaire; *C-SSRS* = Columbia-Suicide Severity Rating Scale; *BDI-II* = Beck Depression Inventory; *BSI-GSI* = Global Severity Index (Brief Symptom Inventory); *DAAPGQ* = Depressive and Anxious Avoidance in Prolonged Grief Questionnaire; *ECR RD8* = Experiences in Close Relationships – Revised (short version); *GCQ* = Grief Cognitions Questionnaire; *ICG-D* = Inventory of Complicated Grief; *MMSE-2* = Mini-Mental-State-Examination; *PCSD* = Perception of Circumstances Surrounding the Death Scale; *PCT* = Present-centered therapy; *PG13+9* = Interview for Prolonged Grief-13, extended version; *PG-CBT* = integrative cognitive behavioral therapy for prolonged grief; *PSQ* = Pain Sensitivity Questionnaire; *SCID-I* = Structured Clinical Interview for DSM-IV Axis-I Disorders; *SOMS-7T* = Screening für Somatoforme Störungen (Screening for Somatoform Disorders); *UGRS* = Utrecht Grief Rumination Scale; *WAI-SR* = Working Alliance Inventory – self-report. *ICG-D is completed before each therapy session

Interested participants, who are potentially eligible (i.e., death of a significant other at least 6 months previously and aged 18 to 75 years), are invited to a first clinical interview. If an individual does not present severe mental symptoms that warrant immediate clinical attention (e.g., acute suicidal ideation assessed using the Columbia-Suicide Severity Rating Scale; CSSRS; [35]) and scores 20 points or more on a screener for PGD symptoms, the ICG-D (Inventory of Complicated Grief; see below), they receive oral and written information about the trial and are invited for baseline assessment. In baseline assessment (after individuals have given their written informed consent), a trained rater assesses PGD diagnosis (PG-13) and comorbidity using the German version of the Structured Clinical Interview for DSM-IV Axis-I Disorders (SCID-I; [36]). In addition, cognitive functioning is tested with the Mini Mental State Examination (MMSE-2; [37]) with individuals aged over 50 years. Self-report measures are given to the participants to be completed at home. If an individual is eligible, randomization is requested of the CTCC before a third appointment. The potential participant is then informed about the randomization result. Thus, the allocation sequence is concealed from the investigators until actual randomization. If an individual does not fulfill the eligibility criteria, or withdraws their consent after randomization, they are counseled about treatment alternatives. Trained raters blinded for treatment allocation conduct the baseline and all further assessments. During treatment, participants complete the PGD screener before each session, while therapists complete a short session screener afterwards, concerning session content including a list of possible serious adverse events. In addition, after sessions 5, 10 and 15, PGD is assessed with the PG-13 and instruments measuring secondary and tertiary outcomes/possible mediators are administered. At post treatment (or in case of dropout if the participant is willing), self-report measures are given to the participant to fill in at home. PGD diagnosis is assessed in a diagnostic interview scheduled after the last treatment session. Follow-up diagnostic takes place 12 months after randomization. See Fig. 2 for an overview of measures and assessment points.

### Interventions

Both interventions are manualized treatments that encompass 20 individual sessions, with the option of four additional sessions to cover special occasions or needs (e.g., another loss or an anniversary). Consequently, the overall treatment dose is flexible from 20 to 24 sessions. In addition, if the patient and therapist feel that the treatment goals have been reached, therapy might be finished earlier. Nonetheless, therapists are asked to stick to the respective treatment manuals concerning content and sequence of sessions. Sessions are scheduled weekly whenever possible, a session lasting 50 min, although some sessions can be combined (100 min). The overall length of treatment is about 6 months.

#### Integrative cognitive behavioral therapy for PGD – PG-CBT

PG-CBT is an integrative but predominantly cognitive behavioral treatment approach that combines classical behavioral methods, like education on how symptoms are perpetuated, homework assignments, exposure and cognitive restructuring with solution-focused, experiential and systemic methods (for an overview see [38]). The initial seven sessions focus on motivation and developing individual goals after further exploration of the patients’ situation and psychoeducation. After teaching relaxation techniques, nine central sessions focus on exposure and cognitive restructuring. Exposure sessions are scheduled to last longer, if necessary, up to 100 min to allow for intense emotional processing. The four final sessions focus on loss integration and future prospects while maintaining a healthy bond to the deceased. In addition to the original manual [39], comorbidity will be specifically addressed throughout the treatment. For example, when educating about PGD, comorbid symptoms (e.g., somatoform pain) are considered in their relation to grief symptoms and this information is integrated into an individualized PGD model.

#### Present-centered therapy – PCT

In terms of session length and number, the PCT manual for PTSD (Shea MT, Bernardy N, Howard J, Key F, Lambert: Present Centered Therapy Manual, PCT. Developed for use in VA CSP-494, treatment of PTSD in women veterans, unpublished) was adapted in cooperation with one of its original authors to PG-CBT. PTC does not include grief-specific behavioral components (e.g., exposure), except for education on grief symptoms and their relation to current problems. Its focus is on the daily monitoring of stressors and problems and on their active mastery. The therapist provides support and an empathic relationship but uses no active interventions except for giving information, pointing out themes, or other ways of fostering functional coping and the client’s problem-solving skills. The first three to four sessions are for establishing a therapeutic relationship and for education on therapy rationale and the interplay of grief symptoms and problems in day-to-day life. A structured daily diary for monitoring these problems is introduced. All following sessions focus on current problems brought forward by the client. The final two sessions are dedicated to closure, focusing on what has been learnt and new aims for the future.

#### Therapists and adherence

Licensed psychotherapists or master’s-level psychologists in advanced postgraduate clinical training administer the treatments. At least two therapists per study center and treatment condition should be recruited. To minimize allegiance bias favoring the experimental treatment, eligible therapists choose for themselves the treatment they want to be trained in after receiving outlines of the two treatment approaches and a summary of their respective efficacy. Therapists then undergo training (2 days) of either PG-CBT (delivered by the first author, RR) or PCT (delivered by one of PCT’s original authors, Tracie Shea). Each therapist will treat one pilot patient under supervision before entering the trial. During the trial, therapists are supervised bi-weekly at the respective study center. In addition, they participate in centralized telephone case consultations. How often therapists participate in supervision will be documented. Treatment sessions will be videotaped. Independent raters will assess treatment fidelity by means of rating randomly chosen treatment tapes.

### Measures

#### Primary outcome

Primary outcome will be the severity score of the Interview for Prolonged Grief-13 (PG-13; [3]) at follow-up, 12 months after randomization. Psychometric evaluation showed good internal consistency (e.g., Cronbach’s alphas from 0.83 to 0.93 in [40]). The PG-13 largely corresponds to the PGD criteria in the upcoming ICD-11, assessing the amount of separation distress (for a diagnosis, the respondent should experience yearning or intense emotional pain at least daily) and nine additional cognitive, emotional, or behavioral grief symptoms (five out of nine should be at least experienced daily). In addition, the respondent must show significant impairment in social, occupational, or other important areas of functioning, for meeting all criteria for a PGD diagnosis. Furthermore, nine more items were added to the German version of the PG-13, resulting in the PG13+9 (Vogel A, Pfoh G, Rosner: PG13+9 [Interview for Prolonged Grief – revised and extended translation of the PG-13], unpublished manuscript; see Additional file 2) in order to encompass all the proposed ICD-11 criteria [2] and the DSM-5 diagnosis “persistent complex bereavement disorder” (PCBD; American Psychiatric Association, [4]). This allows for later estimations of how many participants will fulfill ICD-11 or PCBD criteria, respectively.

#### Secondary outcomes

PGD symptom severity at post treatment will be reported as a secondary outcome. In addition, the following outcomes will be evaluated for both post treatment and follow-up (all assessed with self-report instruments):

- Overall mental health measured with the Global Severity Index (GSI) of the German version of the Brief Symptom Inventory (BSI; [41]), a widely used abbreviated version of the Symptom Checklist-90 – Revised [42]. It targets distress caused by somatic and mental health symptoms in the previous 7 days. The BSI-GSI showed excellent psychometric properties in a German clinical sample, with Cronbach’s α = 0.96 [41]
- Depressive symptoms in the previous 2 weeks, measured with the German revised version of the Beck Depression Inventory (BDI-II; [43]) that showed good Cronbach’s alphas ranging from 0.84 to 0.89 in German clinical and non-clinical samples [44]
- Somatoform symptoms according to the Screening for Somatoform Disorders (SOMS-7T) [45] that assesses somatic symptom severity during the previous 7 days. It is also well-validated for the assessment of change [46] and its internal consistency is excellent, with Cronbach’s α = 0.92

#### Process measures, moderator and mediator variables

Several variables related to coping with loss and grief processing are measured that – on the one hand – could be considered as tertiary outcomes, as they have already been shown to decrease when targeted with grief-specific psychotherapy: grief-related rumination [10], dysfunctional cognitions [24] and grief-related avoidance [24, 47]. On the other hand, these variables might mediate overall grief symptom reduction. In addition, process variables that might shed light on therapeutic process and differential efficacy of both treatments are included as well: therapeutic alliance, intervention-related expectancy, treatment fidelity and therapeutic competence. To monitor the improvement (or worsening) of grief symptoms between sessions, each session starts with the PGD screener (ICG-D, see below). Finally, variables that might moderate treatment success are evaluated, some of them have already been targeted in earlier research (age, time since loss, type of kinship to the deceased and other circumstances of the loss). Others have been less often researched up to now, like the role of comorbidity and somatization in particular and attachment insecurity (see also [48]) or are even completely new in the therapeutic context, like the subjective pain threshold that might be related to the processing of social pain and loss [49]. Demographics and most of the loss-related variables are assessed during the first clinical interview. Treatment fidelity, therapist adherence and competence will be evaluated by independently rated videotapes of randomly selected therapy sessions. Ratings adapted for the respective treatment condition will be developed based on existing rating scales (e.g., [50]). Furthermore, patient compliance (e.g., doing homework assignments or regularly keeping a diary) will be monitored. All other above-mentioned variables will be assessed in self-report:

- Grief symptoms with the German version of the Inventory of Complicated Grief [51], the ICG-D [52]. The ICG-D showed good psychometric properties, with Cronbach’s α = 0.87
- How the loss was experienced with a translation of the short (only four items) Perception of Circumstances Surrounding the Death Scale (PCSD; [53])
- Grief-related rumination; for example, repetitive and recurrent thinking about causes and consequences of the loss, with the Utrecht Grief Rumination Scale (UGRS; [54]); the UGRS’s original version showed good psychometric properties, the German translation is in the process of being validated
- Negative, grief-related cognitions with the Grief Cognitions Questionnaire (GCQ; [55]); the psychometric properties of the original version were good, with adequate convergent and discriminative validity. For the trial, four subscales are used targeting global negative beliefs about self, life and future, as well as about threatening interpretations of one’s own grief reactions. They have been translated into German and are in the process of validation
- Grief-related avoidance with the Depressive and Anxious Avoidance in Prolonged Grief Questionnaire (DAAPGQ; [48]); its two subscales measure avoidance of the reality of the loss (anxious avoidance) and behavioral avoidance and inactivity after loss (depressive avoidance), with good internal consistencies [48, 56]. The German translation will be validated during the trial
- Attachment-related anxiety and avoidance with the short version of the Experiences in Close Relationships – Revised (ECR-RD8; Ehrenthal JC, Zimmermann J, Dinger U, Schauenburg H, Brenk-Franz K, Kirchmann H, et al.: Development and factor structure of a brief screening version of the attachment questionnaire “Experiences in Close Relationships–Revised”, ECR-RD8, in preparation). The unabridged German version [57] is based on the US-American original [58] and showed excellent internal consistencies for the two subscales attachment anxiety and attachment avoidance (Cronbach’s α = 0.91/0.92)
- Pain sensitivity with a subscale of the Pain Sensitivity Questionnaire (PSQ; [59]), the PSQ-minor, which targets pain sensitivity regarding day-to-day situations (e.g., a sunburn or bumping a shin). The PSQ-minor score (Cronbach’s α = 0.81; [59]) was strongly related to subjective pain thresholds in experimental settings [60] and differentiated between a depressive and a healthy sample [61]
- Credibility of both treatments and the participant’s expectations concerning treatment success with the Credibility/Expectancy Questionnaire (CEQ; [62]). A German translation will be adapted for the trial
- Therapeutic alliance with both the therapist and the patient version of the revised short version of the Working Alliance Inventory – Short Revised (WAI-SR; [63]). The German translation of the patient version showed good internal consistencies with Cronbach’s alphas ranging from 0.81 to 0.91 in three clinical samples [64]

### Data management and storage

Data will be collected on paper and then entered in the electronic Case Report Form (eCRF) via remote data entry. Only authorized and trained study personnel receives a login ID in line with their task (e.g. data entry). A study coordinator of the respective study center must authorize the individual eCRF for each enrolled participant and each assessment point. The clinical data management system provided by the CTCC complies with the relevant international standards and has the capability to perform the major data management activities within a consistent, auditable and integrated electronic environment (query management, data entry, data validation and plausibility checks). Only pseudonymized data is transferred to the CTCC via connections secured by SSL technology. Identifying information about participants and administrative forms (e.g., session tapes, informed consents) will be kept in locked cabinets in areas with limited public access or on secured servers for a maximum of 10 years. For long-term storage, the original, pseudonymized data (after database lock) will be stored at the Eichstätt trial center, and will be made available on request to scientific colleagues after publication of the results. In addition, and independently of the trial, study therapies covered by health insurance companies are documented in line with the legal regulations applicable to clinical outpatient centers.

### Statistical analysis

The primary endpoint will be analyzed using a linear mixed-effect model with baseline adjustment, center as random effect, main effects for group and time, a group-by-time interaction term, and a generalized covariance matrix to account for serial dependency among observations [65]. Stratification randomization factors (study center, type of kinship) will be considered. A parameter of interest is the difference in treatment effect estimated as the difference in mean change *μ* between baseline and follow-up assessments after 12 months between the groups (*H*_0_: *μ*_1_ = *μ*_2_; *H*_1_: *μ*_1_ ≠ *μ*_2_). Confidence intervals (95%) for the point estimate for the difference in mean change will be given as well as the standard error. The primary analysis will be done according to the intention-to-treat principle. Missing values, a potential source of bias, will be considered in line with the framework of Rubin [66]. The effects of potentially necessary modeling strategies (i.e., regression computational methods, multiple imputation) for missing values of the primary outcome will be contrasted through sensitivity analyses. A further sensitivity analysis will be based on the per-protocol population (defined as all randomized patients without any major protocol violations). Further analyses will include covariates of prognostic importance in the linear mixed-effect model. For the secondary endpoints, the statistical analysis will be performed as described above. Whenever possible multivariate testing is preferred, especially with closely related predictors (e.g., rumination and negative cognitions). The most promising predictors according to regression analyses will be further evaluated in mediator analysis.

### Monitoring, safety and ethical considerations

The study was planned and is conducted in accordance with the International Council for Harmonisation Guideline for Good Clinical Practice [67]. It has been approved by the Institutional Review Board of the Catholic University Eichstätt-Ingolstadt and their local counterparts at all study centers. Data integrity will be monitored by the CTCC. Study safety will be ensured by monitoring for the incidence of serious adverse events (e.g., suicide attempts, unplanned hospitalizations, occurrence of life-threatening conditions) throughout the treatment phase and at post assessment and follow-up. All such incidents and other aspects of study safety will be regularly reported to an independent Data and Safety Monitoring Board, which provides advice on protocol changes in the event of such incidences, or even on discontinuation of the trial. However, no harm to participants is to be expected. In our pilot trial, there was no clinical significant deterioration during treatment [12]. Face-to-face CBT approaches for PGD have been tested in several trials. The control condition, PCT, has not yet been tested in patients with PGD, but in several studies with patients suffering from PTSD, most of them military (active or veterans) or women who had either experienced childhood sexual abuse or multiple victimization in adulthood. In these trials, PCT was received well, with moderate to high pre- to post-treatment effect sizes. Taken together, both treatments, PG-CBT and PCT, seem well suited and beneficial for the trial participants. Still, psychotherapeutic treatment can be emotionally challenging or distressing and might lead to a temporary worsening of symptoms. In the event of clinical deterioration; for example, if a participant were to develop severe suicidal ideation, adequate treatment would be organized. Although the participant would be excluded from the trial in the event of inpatient treatment longer than 2 weeks, they would be free to continue the treatment anytime, if clinically appropriate. Experienced supervisors are available at all trial sites to decide about necessary measures (e.g., referral to inpatient treatment).

All participants are informed about the study in oral and written form, addressing the trials’ procedures, risks, costs, confidentiality, data storage, and the right to discontinue participation at any time and without giving any reasons. Participants are free to continue treatment with their therapists on quitting the research program. For the sake of data completeness, participants who drop out are asked to continue to participate in assessments if they are willing to give reasons for therapy dropout, but can, of course, decline without any further consequences. Treatment costs are covered by health insurance. Participants receive compensation for travel costs arising from diagnostic, but not therapy sessions. In addition, they will receive a small compensation for taking part in post and follow-up assessment (EUR 20 each).

## Discussion

In this trial, a grief-specific treatment, PG-CBT, is compared to an active, yet unspecific treatment, PCT. While a small pilot trial found that non-specific behavioral activation might suffice to improve PGD [11] and PCT proved quite effective in the treatment of PTSD – which is also a stressor-induced disorder – we still expect PG-CBT, with its integrative cognitive behavioral approach, to be even more effective, as it (1) directly targets grief-related avoidance behavior and dysfunctional beliefs with a wealth of methods, including exposure to foster profound emotional processing and (2) addresses the interplay of grief and comorbid symptoms and somatic complaints, which often accompany prolonged grief. With a planned sample size of more than 200 participants, this multicenter trial is adequately powered for detecting a medium effect size for the comparison between the treatment conditions. In addition, several predictors of treatment outcome (moderator and mediator variables) will be explored, shedding light on the actual process of therapeutic change. Apart from known moderator variables relating to the nature of the loss (e.g., type of kinship) that still need more corroboration and from potential mediator variables pointing to dysfunctional grief processing (e.g., rumination), we also explore newer theories on grief processes (i.e., social pain and its relation to physical pain; see [49]). Special emphasis will be placed on therapeutic process measures, addressing therapeutic alliance throughout the trial as well as therapeutic competence. We hope to detect specific indications for directly targeting prolonged grief and related symptoms in a comprehensive integrative treatment approach (PG-CBT) on the one hand vs. more basically addressing current problems and exclusively focusing on supporting the client and fostering their problem-solving skills (PCT) on the other. The latter might suffice for clients who present with few comorbid disorders or for those who are younger and more resourceful to start with.

The trial has several methodological strengths that will enhance internal validity; for example, block randomization with randomly varying block sizes and stratification performed independently by a CTCC with allocation shortly before enrollment to avoid any kind of selection bias. The primary outcome will be assessed in a structured interview by trained raters who are blind to treatment condition. Both treatment conditions follow structured written manuals, and treatment fidelity will be evaluated together with therapeutic competence and therapists’ participation in supervision. Continuous supervision, locally but also centrally via telephone conference calls, will further ensure treatment fidelity. Unlike in other trials, therapists choose the treatment they feel more comfortable with. This will not only reduce allegiance bias, thereby enhancing the trial design’s internal validity, but will also increase external validity and the clinical relevance of the trial. In clinical practice, therapists are quite free to choose their methods and some do not endorse exposure methods, so PCT might be a viable choice for them. The trial is of clinical relevance in other ways too: the four study centers are part of routine health care in their respective regions and we expect participants to be fairly representative for treatment-seeking patients in Germany. Exclusion criteria are kept to a minimum to further ensure external validity.

The trial limitations might be the following: If PCT succeeds even more than expected, sample size might be too small to detect the hypothesized superiority of PG-CBT. Cell frequencies of the more refined mediator analyses might be low and thus not suffice for substantial conclusions. We use the SCID-I for DSM-IV to assess comorbid disorders as no German version for DSM-5 has been available up to now. Furthermore, some of the instruments used to measure potential mediators and moderators were only translated recently into German, with only pilot validations up to date. Further criticisms might be that the treated disorder in question, PGD, is still not a classifiable mental health disorder, its final definition and symptom criteria still unclear. On the other hand, the diagnosis is a “condition for further study” in DSM-5 (persistent complex bereavement disorder; [4]) and will be included in the forthcoming ICD-11. By adding nine items in the PG-13, we expect to cover all possible future symptom criteria of PGD.

Taken together, the results of this trial will increase knowledge not only about the efficacious treatment of this newly introduced clinical condition but also about its definition and description. We hope that the very conduct of the study will help to disseminate more knowledge about PGD and its treatment in the clinical field.

### Dissemination

The results of the study will be published irrespective of a significant outcome concerning the primary endpoint. They will be disseminated in the following ways. To reach the scientific public, the results will be presented at national and international congresses as well as in publications in peer-reviewed journals, at least two of them open access. To reach stakeholders in public health care and the professional public at large (e.g., primary care providers, psychiatrists and psychotherapists), presentations and workshops will be staged at conferences mainly targeting practitioners. In particular, we hope that the results of this trial will inform the development of treatment guidelines for PGD in Germany. Finally, the interested lay audience (e.g., self-help organizations and Internet groups) will be addressed via interviews in public media or public presentations.

## Trial status

Participant enrollment started in October 2017.

## Additional files

Additional file 1: SPIRIT 2013 Checklist. (PDF 55 kb)

Additional file 2: PG13+9. (PDF 26 kb)

## Abbreviations

BDI-II: Beck Depression Inventory
BSI: Brief Symptom Inventory
CBT: Cognitive behavioral therapy
CEQ: Credibility/Expectancy Questionnaire
CSSRS: Columbia-Suicide Severity Rating Scale
CTCC: Clinical Trials Coordination Center
DAAPGQ: Depressive and Anxious Avoidance in Prolonged Grief Questionnaire
DSM: *Diagnostic and Statistical Manual of Mental Disorders*
eCRF: Electronic Case Report Form
ECR-RD8: short version of the Experiences in Close Relationships – Revised
GCQ: Grief Cognitions Questionnaire
GSI: Global Severity Index
ICD: *International Classification of Diseases*
ICG-D: Inventory of Complicated Grief
MMSE-2: Mini Mental State Examination
PCBD: Persistent complex bereavement disorder
PCSD: Perception of Circumstances Surrounding the Death Scale
PCT: Present-centered therapy
PG13+9: Interview for Prolonged Grief-13, extended version
PG-13: Interview for Prolonged Grief-13
PG-CBT: Integrative cognitive behavioral therapy for prolonged grief
PGD: Prolonged grief disorder
PSQ: Pain Sensitivity Questionnaire
PTSD: Posttraumatic stress disorder
RCT: Randomized controlled trial
SCID-I: Structured Clinical Interview for DSM-IV Axis-I Disorders
SOMS-7T: Screening für Somatoforme Störungen (Screening for Somatoform Disorders)
SPIRIT: Standard Protocol Items: Recommendations for Interventional Trials
UGRS: Utrecht Grief Rumination Scale
WAI-SR: Working Alliance Inventory – Short Revised
