# Supplementary material for: Treating prolonged grief disorder with prolonged grief-specific cognitive behavioral therapy: study protocol for a randomized controlled trial
Source: Trials. 2018 Apr 20;19:241. doi: 10.1186/s13063-018-2618-3 (PMC5910599; doi:10.1186/s13063-018-2618-3)
Supplement: Supplementary file 2 — PG13+9. (PDF 26 kb) [file 13063_2018_2618_MOESM2_ESM.pdf]

Additional File 2:
Erhebungsbogen für anhaltende Trauer (PG13+9)
Erweiterte deutsche Forschungsversion des PG-13

Teil 1: Bitte jeweils eine Antwort pro Frage ankreuzen.

1. Wie oft hatten Sie im letzten Monat Sehnsucht oder starkes Verlangen nach der verstorbenen Person und es ging Ihnen schlecht dabei?

○ Überhaupt nicht (1)
○ Mindestens einmal (2)
○ Mindestens einmal pro Woche (3)
○ Mindestens einmal pro Tag (4)
○ Mehrmals täglich (5)

2. Wie oft hatten Sie im letzten Monat intensive Gefühle von Schmerz, Wehmut oder Trauer in Bezug auf die verstorbene Person?

○ Überhaupt nicht (1)
○ Mindestens einmal (2)
○ Mindestens einmal pro Woche (3)
○ Mindestens einmal pro Tag (4)
○ Mehrmals täglich (5)

3. Falls die Fragen 1 und 2 mindestens einmal mit 4 oder 5 beantwortet wurden: Dauern diese Erfahrungen seit mindestens sechs Monaten?

○ Nein
○ Ja

4. Wie oft im letzten Monat versuchten Sie, die Erinnerung daran, dass die verstorbene Person tatsächlich nicht mehr da ist, von sich zu schieben?

○ Überhaupt nicht (1)
○ Mindestens einmal (2)
○ Mindestens einmal pro Woche (3)
○ Mindestens einmal pro Tag (4)
○ Mehrmals täglich (5)

© Vogel, Pfoh & Rosner, 2017 (Download: http://www.trauma-fortbildung.de/trauerfragebogen)

5. Wie oft im letzten Monat waren Sie bezüglich des Verlustes fassungslos, schockiert oder benommen?

- ○ Überhaupt nicht (1)
- ○ Mindestens einmal (2)
- ○ Mindestens einmal pro Woche (3)
- ○ Mindestens einmal pro Tag (4)
- ○ Mehrmals täglich (5)

<u>Teil 2: Bitte geben Sie für jede Frage an, wie es Ihnen momentan geht (jeweils eine Antwort ankreuzen).</u>

6. Fühlen Sie sich ratlos bezüglich Ihrer Rolle im Leben oder als wüssten Sie nicht mehr, wer Sie sind (z.B. als ob ein Teil Ihrer Selbst gestorben wäre)?

- ○ Überhaupt nicht (1)
- ○ Kaum (2)
- ○ Ein wenig (3)
- ○ Ziemlich (4)
- ○ Sehr (5)

7. Fällt es Ihnen schwer, Ihren Verlust zu akzeptieren?

- ○ Überhaupt nicht (1)
- ○ Kaum (2)
- ○ Ein wenig (3)
- ○ Ziemlich (4)
- ○ Sehr (5)

8. Fällt es Ihnen seit Ihrem Verlust schwer, anderen Menschen zu vertrauen?

- ○ Überhaupt nicht (1)
- ○ Kaum (2)
- ○ Ein wenig (3)
- ○ Ziemlich (4)
- ○ Sehr (5)

9. Sind Sie verbittert über Ihren Verlust?

- ○ Überhaupt nicht (1)
- ○ Kaum (2)
- ○ Ein wenig (3)
- ○ Ziemlich (4)
- ○ Sehr (5)

10. Glauben Sie, dass es jetzt schwierig für Sie ist, Ihr Leben weiterzuführen (z.B. neue Freunde zu finden, neue Interessen zu verfolgen)?

- ○ Überhaupt nicht (1)
- ○ Kaum (2)
- ○ Ein wenig (3)
- ○ Ziemlich (4)
- ○ Sehr (5)

11. Fühlen Sie sich wie betäubt seit Ihrem Verlust?

- ○ Überhaupt nicht (1)
- ○ Kaum (2)
- ○ Ein wenig (3)
- ○ Ziemlich (4)
- ○ Sehr (5)

12. Kommt Ihnen Ihr Leben seit Ihrem Verlust unerfüllt, leer oder bedeutungslos vor?

- ○ Überhaupt nicht (1)
- ○ Kaum (2)
- ○ Ein wenig (3)
- ○ Ziemlich (4)
- ○ Sehr (5)

Teil 3: Bitte eine Antwort ankreuzen.

13. Haben Sie eine deutliche Einschränkung Ihrer Fähigkeiten im sozialen, beruflichen oder in einem anderen Umfeld (z. B. bei der Bewältigung des Haushalts) bemerkt?

- ○ Nein
- ○ Ja

Teil 4: Bitte eine Antwort ankreuzen.

14. Sind Sie so in Gedanken bei der verstorbenen Person, dass es Ihnen schwer fällt, die Dinge zu tun, die Sie normalerweise tun?

- ○ Überhaupt nicht (1)
- ○ Mindestens einmal (2)
- ○ Mindestens einmal pro Woche (3)
- ○ Mindestens einmal pro Tag (4)
- ○ Mehrmals täglich (5)

15. Fühlen Sie sich schuldig in Bezug auf den Verlust?

- ○ Überhaupt nicht (1)
- ○ Kaum (2)
- ○ Ein wenig (3)
- ○ Ziemlich (4)
- ○ Sehr (5)

16. Fällt es Ihnen schwer, sich gut an glückliche Momente mit Ihrer verstorbenen Person zu erinnern?

- ○ Überhaupt nicht (1)
- ○ Kaum (2)
- ○ Ein wenig (3)
- ○ Ziemlich (4)
- ○ Sehr (5)

17. Fällt es Ihnen seit dem Verlust schwer, Glück, Zufriedenheit oder Freude zu erleben?

- ○ Überhaupt nicht (1)
- ○ Kaum (2)
- ○ Ein wenig (3)
- ○ Ziemlich (4)
- ○ Sehr (5)

18. Sind Sie wütend über seinen/ihren Tod?

- ○ Überhaupt nicht (1)
- ○ Kaum (2)
- ○ Ein wenig (3)
- ○ Ziemlich (4)
- ○ Sehr (5)

© Vogel, Pfoh & Rosner, 2017 (Download: http://www.trauma-fortbildung.de/trauerfragebogen)

19. Fällt es Ihnen schwer zu glauben, dass er/sie wirklich tot ist?

- ○ Überhaupt nicht (1)
- ○ Kaum (2)
- ○ Ein wenig (3)
- ○ Ziemlich (4)
- ○ Sehr (5)

20. Fühlen Sie sich einsam, seitdem er/sie gestorben ist?

- ○ Überhaupt nicht (1)
- ○ Kaum (2)
- ○ Ein wenig (3)
- ○ Ziemlich (4)
- ○ Sehr (5)

21. Wären Sie am liebsten tot, um bei der verstorbenen Person sein zu können?

- ○ Überhaupt nicht (1)
- ○ Kaum (2)
- ○ Ein wenig (3)
- ○ Ziemlich (4)
- ○ Sehr (5)

22. Äußert sich Ihre Trauer stärker als Sie es von Menschen in Ihrem Umfeld kennen?

- ○ Nein
- ○ Ja
